# Prospective Evaluation of Safety and Diagnostic Efficacy of Medical Thoracoscopy in Undiagnosed Exudative Pleural Effusion: Experience From a Tuberculosis High-Burden Country

**DOI:** 10.7759/cureus.63517

**Published:** 2024-06-30

**Authors:** Ajoy K Behera, Ranganath Ganga, Vikas Kumar, Dibakar Sahu, Soma S Kiran, Rakesh K Gupta, Amit K Rath, Nitesh Goyal

**Affiliations:** 1 Pulmonary Medicine, All India Institute of Medical Sciences, Raipur, Raipur, IND; 2 Pathology and Laboratory Medicine, All India Institute of Medical Sciences, Raipur, Raipur, IND; 3 Pulmonary Medicine, SCB (Srirama Chandra Bhanja) Medical College and Hospital, Cuttack, IND

**Keywords:** non-small cell lung cancer, pleural biopsy, pleural tuberculosis, medical thoracoscopy, malignant pleural effusion, tuberculous pleural effusion

## Abstract

Introduction: Pleural effusion is due to the pathological accumulation of pleural fluid in the pleural space, 25%-30% of which may remain undiagnosed despite the combination of biochemical, microbiological, and pathological tests and closed pleural biopsy. Medical thoracoscopy may help physicians diagnose such cases. We aimed to study the diagnostic yield of medical thoracoscopy in patients with undiagnosed exudative pleural effusion and assess the safety profile of the medical thoracoscopy.

Methodology: A cross-sectional descriptive study was conducted on 105 patients with undiagnosed pleural effusion. Medical thoracoscopy was performed using an Olympus semi-rigid thoracoscope (LTF 160 Evis Pleurovideoscope, Japan) as per standard protocol. Multiple pleural biopsies were taken and sent for histopathology examination, NAAT (nucleic acid amplification test), and MGIT (mycobacteria growth indicator tube). Post-procedure, the patients were evaluated for any complications.

Results: A total of 105 patients were enrolled in the study. The mean ± SD age was 55.1 ± 13.6 years. Sixty-three (60%) patients were males. The diagnostic utility of medical thoracoscopy was found in 94 (89.5%) patients. The diagnosis of tuberculosis (TB) was made in 34 (32.3%) patients, and 48 (45.7%) patients were diagnosed with malignant pleural effusion. Adenocarcinoma of the lung was the most common malignancy diagnosed (32 patients, 66.6%). Five (5.31%) patients had dual etiology of pleural effusion: tubercular and malignancy. The most common complication was chest pain following the procedure (99.4%). One patient developed pneumomediastinum and was managed conservatively. There were no major adverse events after the procedure.

Conclusions: Medical thoracoscopy has a high diagnostic yield and favorable safety profile with minimal complications. Excessive reliance on the level of ADA (adenosine deaminase) may further delay the diagnosis. Dual etiologies like TB coexisting with malignancy should be considered in TB high-burden countries.

## Introduction

Pleural effusion is a pathological accumulation of fluid in the pleural space and is common worldwide. The causes of pleural effusion vary widely, ranging from viral pleuritis to malignancy [1]. The first step of evaluation is to determine whether pleural fluid is transudative or exudative using Light's criteria [2,3]. Around 25%-40% of patients with exudative pleural effusion remain undiagnosed after pleural fluid cytology and biochemical evaluation, along with radiological evaluation of the thorax, thus necessitating the need for pleural biopsy [4,5]. Pleural biopsy can be done using a closed pleural biopsy needle, ultrasonography (USG) or CT-guided pleural biopsy, medical thoracoscopy (MT), and video-assisted thoracoscopic surgery (VATS) [6].

The British Thoracic Society guideline recommends thoracoscopic pleural biopsy as the modality of choice for undiagnosed pleural effusion [7]. A recent meta-analysis found closed pleural biopsy to be inferior to MT for the diagnosis of malignant pleural effusions. The sensitivity and specificity in the closed pleural biopsy group were 77% and 99%, while those of MT were 93% and 100%, respectively [8]. The diagnostic efficacy of MT in undiagnosed pleural effusion varies from 70% to 100% [9-11]. Pleural biopsy CBNAAT (cartridge-based nucleic acid amplification test) provided a higher yield than pleural fluid culture and improved yield compared with closed pleural biopsy [12]. These studies also concluded that semi-rigid thoracoscopy was safe and well tolerated by the patients with good diagnostic yield.

The aim of our study was to assess the safety and diagnostic yield of MT in patients with undiagnosed exudative pleural effusion.

## Materials and methods

This cross-sectional descriptive study was conducted in the Department of Pulmonary, Critical Care, and Sleep Medicine, All India Institute of Medical Sciences Raipur. The study was approved by the institute ethics committee by letter no. AIIMSRPR/IEC/2022/1056. The study was conducted from March 2022 to August 2023. The sample size was calculated based on the prevalence of 25-40%. We recruited 105 consecutive patients with undiagnosed exudative pleural effusion. These patients underwent an MT.

Definition of undiagnosed exudative pleural effusion

Pleural fluid was defined by Light's criteria as exudative, with sterile bacterial culture, negative *Mycobacterium tuberculosis* (MTB) acid-fast bacilli (AFB) staining, and CBNAAT with adenosine deaminase levels less than 40 IU/L [13]. Additionally, three consecutive samples for cytology were negative for malignant cells. Patients with ADA levels greater than 40 IU/L but with clinical discordance for tuberculosis (TB) were also included.

Inclusion criteria

Patients aged over 18 years with undiagnosed exudative pleural effusion as per the study definition were included.

Exclusion criteria

Pregnant females, patients not willing to undergo MT, and those with contraindications to thoracoscopy (lack of pleural space due to pleural thickening, previous pleurodesis, refractory cough, severe hypoxemia, unstable hemodynamic parameters, poor general health status, and inability to lie down).

The enrolled patients underwent a detailed history and examination. Complete blood counts, coagulation profiles, and viral markers were done before the procedure. Point-of-care ultrasound of the chest was used to assess the volume of pleural effusion and the status of the underlying lung and to confirm the site for thoracoscopy.

MT procedure

MT was performed after overnight fasting in the pulmonary intervention suite using an Olympus semi-rigid thoracoscope (LTF 160 Evis Pleurovideoscope, Japan). The patient was placed in a lateral decubitus position with the pleural effusion in the upward position. The entire hemithorax was cleaned with povidone-iodine and propyl alcohol and draped under all aseptic precautions. The chosen site was infiltrated with 2% lignocaine (usually 10 mL, with the maximum dose not exceeding 3 mg/kg). A linear incision of approximately 1 cm was made to expose the underlying subcutaneous plane, and blunt dissection was done to enter the pleural space. A thoracoscope was introduced through the trocar placed into the dissection site.

Pleural fluid was aspirated. The costal pleura, diaphragmatic, and visceral pleura surfaces were examined. Abnormalities such as pleural bands (thick, thin, or both), pleural nodules/mass lesions, and their distribution on parietal and visceral pleural surfaces were noted. Multiple pleural biopsies (8 to 12 samples) were taken using 2.8 mm diameter hot biopsy forceps (FD-7C-1, hot biopsy forceps, Olympus, Japan). Heart rate, blood pressure, electrocardiography, and oxygen saturation were monitored continuously during the procedure and for two hours post-MT. After the procedure, an intercostal tube (size 24 F or 28 F) was inserted and secured using non-absorbable sutures. The patient was evaluated for any post-procedural complications during the hospital stay. Pain assessment was done using the VAS (visual analogue scale) [14]. Biopsy samples were collected in formalin (for histopathological examination) and saline (for mycobacterial cultures and nucleic acid amplification test (NAAT)).

Diagnostic criteria

The diagnostic criteria included the following: (1) malignancy: malignancy confirmed histopathologically on a pleural biopsy specimen; (2) tuberculosis: the biopsy specimen showed granulomatous inflammation or was positive for AFB stain, NAAT, or liquid culture for mycobacteria, or chronic pleuritis on biopsy with clinical features suggestive of TB, and improvement upon starting anti-tubercular treatment; (3) nondiagnostic: pleural biopsy showed features of nonspecific pleuritis or normal pleura.

Statistical analysis

Information about the included clinical-demographic profile, pleural fluid analysis, radiological findings, and thoracoscopy findings was entered into a Microsoft Excel sheet (Microsoft Corporation, Redmond, Washington) and cross-checked for duplicate and invalid entries. Analysis was done using IBM SPSS Statistics for Windows, Version 23 (Released 2015; IBM Corp., Armonk, New York). Simple descriptive tabulation and cross-tabulation were drawn. Continuous variables were expressed as mean or median. Categorical variables were expressed as percentages. Sensitivity, specificity, positive predictive value, and negative predictive value were calculated for MT. The STARD statement (Standards for Reporting of Diagnostic Accuracy Studies) was used to complete diagnostic accuracy reports [15,16].

## Results

**Figure 1:**
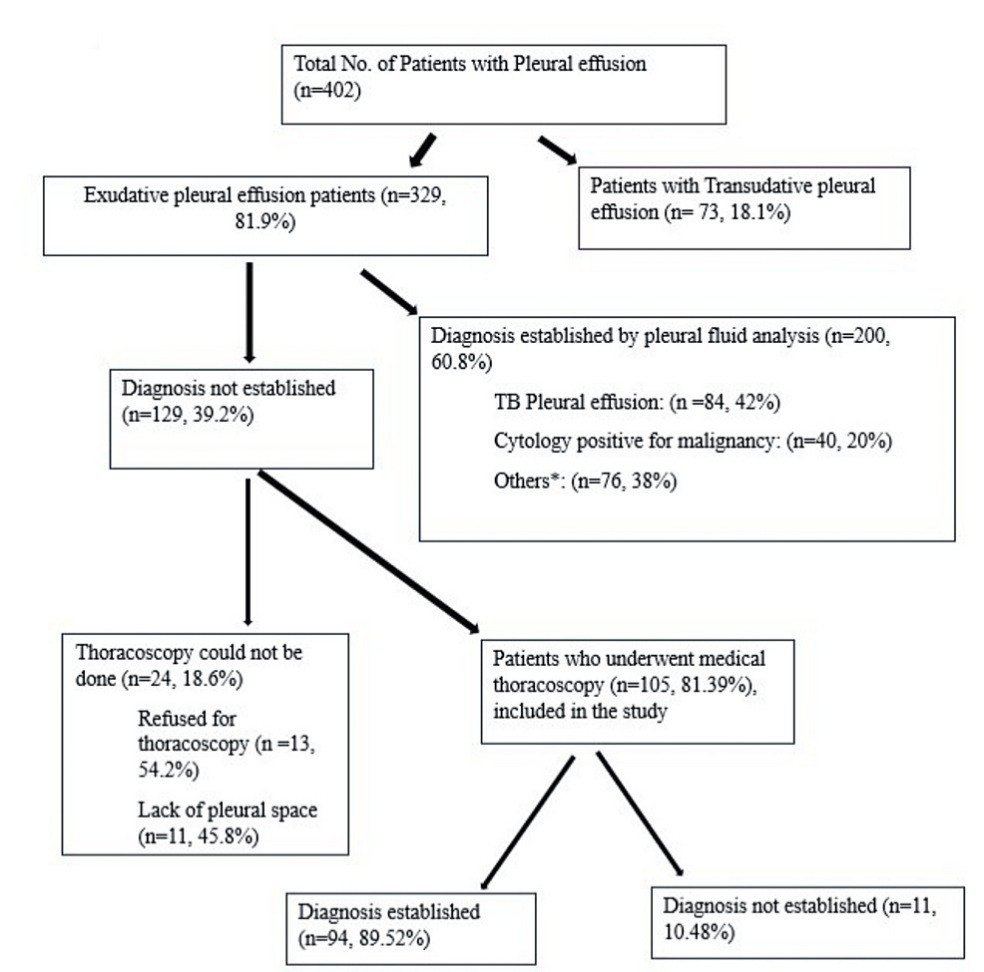
Selection of patients in the study *Others: parapneumonic effusions (50.25%), pancreatitis (10.5%), rheumatoid arthritis (5.25%), hypothyroidism (5.25%), pulmonary embolism (4.2%), esophageal tear (2.1%).

We screened 402 patients, of which 105 were enrolled in the study as depicted in Figure 1. The demographic and pleural fluid characteristics of the study population are depicted in Table 1. Exudative pleural effusion was present in 329 patients (81.9%), and undiagnosed exudative pleural effusion was present in 129 patients (39.2%).

**Table 1:** Demographic and pleural fluid characteristics of the study population (n =105) *Socioeconomic class as per the modified Kuppuswamy scale [17]. SD: standard deviation; BMI: body mass index; TLC: total leukocyte count; IQR: interquartile range; LDH: lactate dehydrogenase; ADA: adenosine deaminase.

Parameter	Values
Age (mean ± SD), years	55.1 ± 13.6
Males, n (%)	63 (60%)
Smokers, n (%)	16 (15.2%)
Lower socioeconomic class, n (%)*	78 (74.3%)
BMI (mean ± SD), kg/m^2^	20.4 ± 2.26
Massive pleural effusion, n (%)	55 (52.38%)
Side of pleural effusion (right), n (%)	54 (51.42%)
TLC, median (IQR)	250 (95-600)
Lymphocytic predominance, n (%)	96 (91.42%)
LDH, median (IQR), U/L	453 (252-634)
Protein (mean ± SD), g/dL	4.68 ± 0.95
ADA (mean ± SD), U/L	30.3 ± 17.7
Glucose (mean ± SD), mg/dL	92.4 ± 42.4

Fifty-five (52.38%) patients presented with massive pleural effusion. Right-sided pleural effusion was found in 54 (51.42%) patients. Ninety-six (91.42%) patients had lymphocytic predominant pleural effusion.

**Figure 2:**
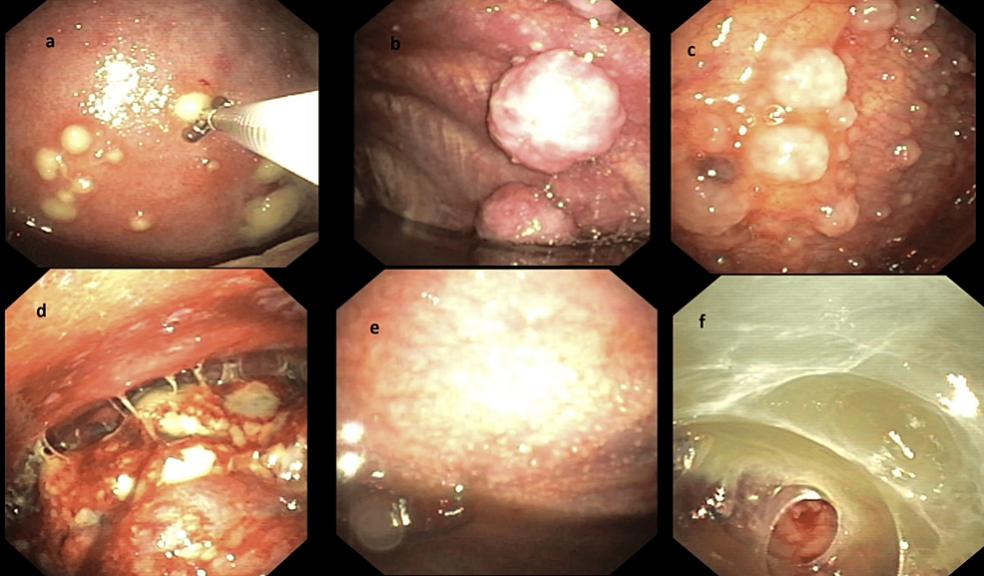
Macroscopic appearance of the pleural surface during thoracoscopy Thoracoscopic findings and histopathology of biopsy: a - small yellowish nodule (adenocarcinoma); b - large nodules (adenocarcinoma); c - grapes-like cluster of nodules (mesothelioma); d - cauliflower-like growth (poorly differentiated adenosquamous carcinoma); e - sago grain appearance (tubercular granulomatous inflammation); f - dense adhesions (chronic nonspecific inflammation).

All 105 enrolled patients underwent contrast-enhanced CT of the chest. It showed pleural effusion only in 53 (50.4%) patients, pleural nodularity in 17 (16.19%) patients, lung mass with pleural effusion in 20 (19.04%) patients, and mediastinal lymphadenopathy in 22 (20.9%) patients. On thoracoscopy, pleural nodules were the most common finding in 79 (75.2%) patients, followed by adhesions in 14 (13.3%), both nodules and adhesions in 6 (5.7%), and normal pleura in 6 (5.7%) patients. Figure 2 reveals various thoracoscopic findings.

Of the 105 patients enrolled, thoracoscopy-guided pleural biopsy yielded a diagnosis in 94 (89.5%, 95% CI: 0.82-0.94). The overall sensitivity, specificity, positive predictive value, and negative predictive value of MT were 90.3%, 100%, 100%, and 9.09%, respectively.

The details of the diagnosis are given in Table 2. Ten (9.52%) patients had features of non-specific pleuritis. These patients were started on anti-tubercular treatment. On follow-up, clinic-radiological improvement was noted. In the remaining patients, no other definitive features were found on biopsy.

**Table 2:** Etiologies of pleural effusion established by medical thoracoscopic biopsy in the study (n=94) *Among five patients, four patients had adenocarcinoma lung with tuberculosis, and one had plasma cell leukemia with tuberculosis.

Etiology of pleural effusion	N (%)
Tubercular pleural effusion	34 (36.17%)
Adenocarcinoma lung	32 (32.04%)
Metastatic breast cancer	6 (6.38%)
Dual etiologies (tuberculosis plus malignancy)*	5 (5.31%)
Malignant mesothelioma	3 (3.19%)
Bacterial empyema	3 (3.19%)
Metastatic adenocarcinoma (from the gastrointestinal tract)	2 (2.12%)
Fungal empyema	1 (1.06%)
Thoracic endometriosis	1 (1.06%)
Systemic lupus erythematosus	1 (1.06%)
Pleural neurofibromatosis	1 (1.06%)
Squamous cell cancer lung	1 (1.06%)
Adenosquamous cell cancer lung	1 (1.06%)
Metastatic endometrial cancer	1 (1.06%)
Metastatic giant cell tumor	1 (1.06%)
Metastatic synovial cell sarcoma	1 (1.06%)

The most common cause of undiagnosed exudative pleural effusion was malignancy. The most common cause of malignant pleural effusion was adenocarcinoma of the lung (32, 66.6%). Six (12.5%) patients had metastasis from breast cancer. Five (10.4%) patients had metastasis from other sites (two from metastatic adenocarcinoma of the gastrointestinal tract, one from endometrial cancer, and one each from giant cell tumor of the femur and synovial cell sarcoma of the tibia). Three patients had malignant mesothelioma. One patient had squamous cell cancer of the lung, and one had adenosquamous cell cancer of the lung. Tubercular pleural effusion was the second most common cause found (34, 36.17%).

Five (4.76%) patients had dual etiologies (TB with malignancy). Among these five patients, four had adenocarcinoma of the lung with TB, and one had plasma cell leukemia with TB. Four patients had growth of *Mycobacterium tuberculosis* (MTB) on mycobacteria growth indicator tube (MGIT), and one patient was diagnosed with polydrug-resistant TB along with adenocarcinoma of the lung. MTB detection via NAAT was seen in only one patient.

Procedure-related complications

One hundred four (99.4%) patients experienced chest pain following the procedure. Ninety patients (86.5%) had mild chest pain, and 14 (13.5%) patients had moderate chest pain as per the VAS scale [14]. The pain subsided after the administration of analgesics. Seventeen (16.1%) patients had minor bleeding not requiring specific intervention such as hypotension or blood transfusion. One (0.9%) patient developed pneumomediastinum, which resolved spontaneously after five days without any surgical intervention. The median hospital stay was 10 days, IQR (7-12) days, and there was no prolonged hospital stay due to major complications after the procedure. Overall, the patients tolerated the procedure well without any serious adverse events.

## Discussion

Undiagnosed exudative pleural effusion is a challenge to respiratory physicians in everyday practice. Various methods have been used to evaluate these patients, including closed pleural biopsy, rigid thoracoscopy, and MT [7]. Closed pleural biopsy is inferior to semi-rigid thoracoscopy for the diagnosis of malignant diseases [8]. Therefore, MT helps establish the diagnosis of pleural effusion across socioeconomic groups [17,18]. We found a diagnostic yield of 89.5% (95% CI: 0.82-0.94) for MT in these patients with undiagnosed exudative pleural effusion. Rare diagnoses like plasma cell leukemia, pleural neurofibromatosis, and thoracic endometriosis were made via thoracoscopy-guided biopsy in our study. The range of diagnostic yield in other studies evaluating exudative pleural effusions is from 66 to 100%, and a comparison of yield is shown in Table 3.

**Table 3:** Diagnostic yield of medical thoracoscopy in previous studies as compared to our study.

Study	Study design	Patients (n)	Diagnostic yield (%)
Rai et al. [9]	Retrospective	76	86.8
Agarwal et al. [10]	Prospective	19	69
Thangakunam et al. [11]	Retrospective	21	66.7
Valsecchi et al. [18]	Retrospective	2752	71
Prabhu et al. [19]	Prospective	68	97
Dhooria et al. [20]	Prospective	45	73.3
Wang et al. [21]	Prospective	833	92.6
Maturu et al. [22]	Retrospective	264	94
Nattusamy et al. [23]	Prospective	48	66.7
Patil et al. [24]	Prospective	129	85.2
Kuwal et al. [25]	Prospective	55	83.64
Our study	Prospective	105	89.5

Kuwal et al. found that MT had a sensitivity of 93.88% and a specificity of 100% [25]. In a meta-analysis, Agarwal et al. reported the sensitivity and specificity of MT for the diagnosis of pleural effusion as 91% and 100%, respectively [26]. Mohan et al. found the sensitivity and specificity of MT to be 97% and 100% [27]. The results of our study are comparable with these studies.

Dual etiologies

Five (4.76%) patients had dual etiologies (TB with malignancy), of which four had adenocarcinoma of the lung with tuberculosis and one had plasma cell leukemia with tuberculosis.

Microbiological yield

Four patients had growth of MTB on MGIT, and one patient was diagnosed with polydrug-resistant TB on MTB liquid culture along with adenocarcinoma of the lung. NAAT for MTB was positive in one patient. As per a recent systematic review, TB was found to be a new carcinogenic agent for lung cancer [28].

Complications and safety profile

Of the 105 patients, 99.4% experienced chest pain following the procedure. Among them, 86.5% had mild chest pain, while 13.5% reported moderate chest pain according to the VAS scale. The pain subsided after taking analgesics. Similarly, Wang et al. also reported transient chest pain in 44% of patients following the procedure [21]. Kuwal et al. reported chest pain among 20% of the patients who underwent MT [25]. However, the proportion of patients who experienced chest pain was higher in our study. The differences could be due to variations in the use of sedatives and analgesics during the procedure. In our study, 16.1% of patients had minor bleeding following MT but required specific interventions. Post-procedure bleeding in ICD was also reported in other studies [19,25]. One patient (0.9%) developed pneumomediastinum, which resolved spontaneously after five days of oxygen therapy. The median hospital stay was 10 days (IQR: 7-12). There was no procedure-related prolonged hospital stay, major complications, or mortality in the study. In a meta-analysis by Agarwal et al., no major complications were found post-procedure [26]. Similarly, Mohan et al. did not report major complications of the procedure in a systematic review of four studies [27].

Strengths and weaknesses of our study

The strength of our study was the development of an algorithm to categorize patients with undiagnosed pleural effusion. In all patients, pleural biopsy samples were sent for NAAT, mycobacterial liquid culture, and histopathology. Dual aetiologies were found only in our study, a new finding not reported in previous studies. There are certain limitations to our study. It was a single-center hospital-based study prone to bias. No direct head-to-head comparison was done with other modalities (closed pleural biopsy, ultrasound/CT-guided pleural biopsy). Long-term follow-up of patients with non-specific pleuritis was not done. Sequential pain monitoring (using the VAS) was not performed. Based on the results of the study, we recommend that thoracoscopic pleural biopsy is a safe and useful procedure to establish a diagnosis in patients with exudative pleural effusion.

## Conclusions

Our study concluded that MT-guided pleural biopsy has a good diagnostic yield with a favorable safety profile and should be used more frequently in patients with undiagnosed pleural effusion. This approach can reduce reliance on surgical procedures, thereby lowering economic burden and morbidity. Dual etiologies (TB and malignancy) should not be missed in a high TB endemic country like India.

## References

[1] Jany B, Welte T (2019). Pleural effusion in adults-etiology, diagnosis, and treatment. Dtsch Arztebl Int.

[2] Light RW, Macgregor MI, Luchsinger PC, Ball WC Jr (1972). Pleural effusions: the diagnostic separation of transudates and exudates. Ann Intern Med.

[3] Maskell NA, Butland RJ (2003). BTS guidelines for the investigation of a unilateral pleural effusion in adults. Thorax.

[4] Poe RH, Israel RH, Utell MJ, Hall WJ, Greenblatt DW, Kallay MC (1984). Sensitivity, specificity, and predictive values of closed pleural biopsy. Arch Intern Med.

[5] Prakash UB, Reiman HM (1985). Comparison of needle biopsy with cytologic analysis for the evaluation of pleural effusion: analysis of 414 cases. Mayo Clin Proc.

[6] Beaudoin S, Gonzalez AV (2018). Evaluation of the patient with pleural effusion. CMAJ.

[7] Roberts ME, Rahman NM, Maskell NA (2023). British Thoracic Society guideline for pleural disease. Thorax.

[8] Wei Y, Shen K, Lv T (2020). Comparison between closed pleural biopsy and medical thoracoscopy for the diagnosis of undiagnosed exudative pleural effusions: a systematic review and meta-analysis. Transl Lung Cancer Res.

[9] Rai DK, Niwari LN, Karmakar S, Sharma S (2021). Diagnostic yield of semi rigid thoracoscopy in unexplained exudative pleural effusion - experience from tertiary care hospital of east India. Indian J Tuberc.

[10] Christopher DJ, Gupta R, Thangakunam B (2024). Pleural effusion guidelines from ICS and NCCP Section 1: basic principles, laboratory tests and pleural procedures. Lung India.

[11] Mishra AK, Verma SK, Kant S (2016). A study to compare the diagnostic efficacy of closed pleural biopsy with that of the thoracoscopic guided pleural biopsy in patients of pleural effusion. South Asian J Cancer.

[12] Christopher DJ, Dinakaran S, Gupta R, James P, Isaac B, Thangakunam B (2018). Thoracoscopic pleural biopsy improves yield of Xpert MTB/RIF for diagnosis of pleural tuberculosis. Respirology.

[13] Sharma SK, Ryan H, Khaparde S (2017). Index-TB guidelines: Guidelines on extrapulmonary tuberculosis for India. Indian J Med Res.

[14] McCormack HM, Horne DJ, Sheather S (1988). Clinical applications of visual analogue scales: a critical review. Psychol Med.

[15] Bossuyt PM, Reitsma JB, Bruns DE (2015). STARD 2015: an updated list of essential items for reporting diagnostic accuracy studies. BMJ.

[16] Cohen JF, Korevaar DA, Altman DG (2016). STARD 2015 guidelines for reporting diagnostic accuracy studies: explanation and elaboration. BMJ Open.

[17] Ananthan VA (2021). Modified Kuppuswamy scale for socioeconomic status of the Indian family - update based on New CPI (IW) series from September 2020. J Family Med Prim Care.

[18] Valsecchi A, Arondi S, Marchetti G (2016). Medical thoracoscopy: analysis on diagnostic yield through 30 years of experience. Ann Thorac Med.

[19] Prabhu VG, Narasimhan R (2012). The role of pleuroscopy in undiagnosed exudative pleural effusion. Lung India.

[20] Dhooria S, Singh N, Aggarwal AN, Gupta D, Agarwal R (2014). A randomized trial comparing the diagnostic yield of rigid and semirigid thoracoscopy in undiagnosed pleural effusions. Respir Care.

[21] Wang XJ, Yang Y, Wang Z (2015). Efficacy and safety of diagnostic thoracoscopy in undiagnosed pleural effusions. Respiration.

[22] Maturu VN, Dhooria S, Bal A (2015). Role of medical thoracoscopy and closed-blind pleural biopsy in undiagnosed exudative pleural effusions: a single-center experience of 348 patients. J Bronchology Interv Pulmonol.

[23] Nattusamy L, Madan K, Mohan A (2015). Utility of semi-rigid thoracoscopy in undiagnosed exudative pleural effusion. Lung India.

[24] Patil CB, Dixit R, Gupta R, Gupta N, Indushekar V (2016). Thoracoscopic evaluation of 129 cases having undiagnosed exudative pleural effusions. Lung India.

[25] Kuwal A, Advani M, Dutt N, Saini S, Singh S (2021). Diagnostic accuracy of semirigid thoracoscopy in exudative pleural effusions and relationship of thoracoscopic findings with probability of malignant diagnosis. Monaldi Arch Chest Dis.

[26] Agarwal R, Aggarwal AN, Gupta D (2013). Diagnostic accuracy and safety of semirigid thoracoscopy in exudative pleural effusions: a meta-analysis. Chest.

[27] Mohan A, Chandra S, Agarwal D, Naik S, Munavvar M (2010). Utility of semirigid thoracoscopy in the diagnosis of pleural effusions: a systematic review. J Bronchology Interv Pulmonol.

[28] Hwang SY, Kim JY, Lee HS (2022). Pulmonary tuberculosis and risk of lung cancer: a systematic review and meta-analysis. J Clin Med.

